# Lipidomics of the brain, retina, and biofluids: from the biological landscape to potential clinical application in schizophrenia

**DOI:** 10.1038/s41398-020-01080-1

**Published:** 2020-11-09

**Authors:** Chuanjun Zhuo, Weihong Hou, Hongjun Tian, Lina Wang, Ranli Li

**Affiliations:** 1 Department of Psychiatry (RTNBP_Lab), Tianjin Fourth Center Hospital, Tianjin, 300140 China; 2 Department of Psychiatry (PNGC-Lab), 2 Tianjin Anding Hospital, Tianjin, 300222 China; 3 Department of Biochemistry and Molecular Biology, Zhengzhou University, Zhengzhou, Henan 450001 China

**Keywords:** Physiology, Schizophrenia

## Abstract

Schizophrenia is a serious neuropsychiatric disorder, yet a clear pathophysiology has not been identified. To date, neither the objective biomarkers for diagnosis nor specific medications for the treatment of schizophrenia are clinically satisfactory. It is well accepted that lipids are essential to maintain the normal structure and function of neurons in the brain and that abnormalities in neuronal lipids are associated with abnormal neurodevelopment in schizophrenia. However, lipids and lipid-like molecules have been largely unexplored in contrast to proteins and their genes in schizophrenia. Compared with the gene- and protein-centric approaches, lipidomics is a recently emerged and rapidly evolving research field with particular importance for the study of neuropsychiatric disorders such as schizophrenia, in which even subtle aberrant alterations in the lipid composition and concentration of the neurons may disrupt brain functioning. In this review, we aimed to highlight the lipidomics of the brain, retina, and biofluids in both human and animal studies, discuss aberrant lipid alterations in correlation with schizophrenia, and propose future directions from the biological landscape towards potential clinical applications in schizophrenia. Recent studies are in support of the concept that aberrations in some lipid species [e.g. phospholipids, polyunsaturated fatty acids (PUFAs)] lead to structural alterations and, in turn, impairments in the biological function of membrane-bound proteins, the disruption of cell signaling molecule accessibility, and the dysfunction of neurotransmitter systems. In addition, abnormal lipidome alterations in biofluids are linked to schizophrenia, and thus they hold promise in the discovery of biomarkers for the diagnosis of schizophrenia.

## Introduction

Schizophrenia is a complex, devastating chronic neuropsychiatric disorder that affects ~1% of the population across the world^1^. Patients with schizophrenia usually experience delusions and hallucinations, social deficits, cognitive dysfunction, and apathy; as such, this severe disorder imposes a tremendous burden on the affected family and the society^1–3^. The pathophysiology of schizophrenia has not been clearly identified. Thus far, neither objective biomarkers for diagnosis nor specific medications for the treatment of schizophrenia are clinically satisfactory. Thus, it is necessary to decode the mechanisms underlying this disorder and to identify objective biomarkers for the diagnosis of schizophrenia^2,3^.

Lipids are essential for life, and ~100,000 different lipid species can be found in the lipidome of humans, suggesting greater diversity than proteins^4,5^. Lipids are known to have structural roles, beyond which they act to participate in cellular transport, energy storage, and cellular signaling^5^. Furthermore, lipids have been shown to function as modulators in the regulation of membrane-bound proteins, including ion channels; therefore, their alterations affect the function of these ion channels^5^. The past decade has witnessed substantial progress in the research field of lipidomics, including mass spectrometry (MS) technology as well as bioinformatics software for lipidomics, which have underpinned the fast evolution of the research field of lipidomics. As a relatively new research field, the number of published studies in lipidomics is much lower than that in genomics and proteomics. For example, a recent literature search for “Lipidomics” in PubMed (December 2019) yielded 9146 publications, considerable lower than the 355,316 and 132,662 publications for “Genomics” and “Proteomics”, respectively. This represents a huge opportunity for researchers to acquire new knowledge about lipids related to both health and diseases, including schizophrenia. Notably, lipids play critical roles in the maintenance of the normal structure and function of neurons in the brain, and abnormalities in neuronal lipids are strongly correlated with abnormal neurodevelopment in patients with schizophrenia^6–14^. However, lipids and lipid-like molecules remain largely unexplored, in comparison with proteins and their genes in schizophrenia. In contrast to the gene- and protein-centric approaches, lipidomics is a new research field that has recently emerged and is rapidly evolving. This approach appears to be particularly important for the study of neuropsychiatric disorders like schizophrenia, as subtle aberrant alterations in lipids, in terms of both the composition and concentration of neurons, may disrupt brain functioning.

In this review, we aimed to appraise the knowledge acquired through conducting lipidomics studies in the brain, retina, and biofluids in both humans and animals, with a focus on aberrant lipid alterations in correlation with schizophrenia and to evaluate the progress made in understanding the potential roles for lipids in schizophrenia. We also discuss the limitations and challenges and propose future directions from the biological landscape towards potential clinical applications in schizophrenia. Recent findings support the concept that aberrations in some lipid species [e.g. phospholipids, polyunsaturated fatty acids (PUFAs)] lead to structural alterations and, in turn, impairments in the biological function of proteins bound to the membrane, disruption of cell signaling molecule accessibility, and dysfunctions in neurotransmitter systems (e.g. dopamine). In addition, abnormal lipidome alterations in biofluids are linked to schizophrenia, and thus they hold promise in the discovery of biomarkers for the diagnosis of the disorder.

## Lipidomics approaches

### Conventional lipidomics approaches

Lipidomics is a fast growing research field with increasing studies showing the importance of lipids in health and disease, shedding light on the complex functions of lipids. MS is a conventional tool of choice for the analysis of lipidomes in cells and tissues and has been the main catalyst in driving the field of lipidomics forward. There are two major approaches in MS: direct injection and liquid chromatography (LC)–coupled tandem MS (LC-MS/MS). In direct injection “shotgun lipidomics”, lipid extracts are directly infused into the mass spectrometer without prior separation using chromatography^15,16^. Due to the unique ionization properties of the lipid species in acidic or basic conditions, a large number of complex lipid species can be quickly detected and identified using direct injection “shotgun lipidomics”. Direct injection MS has a major advantage in that only one internal standard per class is able to correct for a given class of lipids, as the lipids are ionized under the same conditions as well as at the same time, which makes quantitation of the lipids simpler and more reliable.

However, several limitations have been noted for shotgun lipidomics, including challenges in resolving lipids with very low abundance and isobaric species^17^. Recent advances have been made in MS‐based lipidomics, allowing access to greater levels of molecular information in lipidomics experiments. These advancements will form the main piece in the puzzle as the field of lipidomics moves towards the characterization of lipids at the molecular level. Lipids are initially separated through high-pressure liquid chromatography (HPLC) in LC-MS/MS, and are further separated, fragmented, and identified in highly sensitive tandem MS (MS/MS). As such, the limitation, namely the ion suppression effects, of direct injection “shotgun lipidomics” can be avoided. Reverse-phase HPLC in combination with MS has been widely used for non-targeted lipidomics.

### Single-organelle lipidomics

The conventional lipidomics approaches, such as direct injection and LC-MS/MS, have limitations. Lipid extraction is usually needed for subsequent analysis, and conventional tools for lipidomics are unable to probe the lipid distribution or study the lipid species in specific cells until lipid extraction is performed. Another limitation is that conventional tools largely rely on lipid extraction from a relatively large amount of samples in cells or tissues, which may average the data and mask cell-to-cell variations.

Recent advances have been made in the development of a new tool, allowing the use of single organelles with Raman microscopy (micro-Raman) assays^18^. Most recently, Lita et al. reported a micro-Raman assay that is applied to measure the intracellular lipid composition, lipidome hallmarks-lipid concentration, cis/trans isomer ratio, unsaturation levels, and the levels of cholesterol and sphingolipids in live cells at a sub-micrometer resolution. It may merit attention that single-organelle lipidomics using a micro-Raman assay is sufficient for profiling the subcellular structures. Furthermore, this newly developed approach in combination with the use of biomolecular component analysis software has provided a platform for both quantitative data analysis and lipid profiling in subcellular structures. This new approach is expected to enhance the ability of Raman-based lipidomics for single-organelle analysis of either live or fixed cells. Raman-based lipidomics represents an exciting approach that also allows for the measurement of organellar lipid heterogeneity and opens a new path for quantitative analysis of phenotypic variability.

## Aberrant lipidome in schizophrenia as revealed by lipidomics

### Lipidomics of the different regions in the brain reveals aberrant lipidome in relation to schizophrenia

Lipidomics of the different regions in the brain has revealed aberrant lipidome composition changes, which are accompanied by variations in genes related to lipids and alterations in schizophrenia-associated pathways^6–14^. Recently, Yu et al.^14^ evaluated the lipidomics of the dorsolateral prefrontal cortex (PFC) gray matter in cognitively healthy individuals (*n* = 396) and patients with cognitive disorders, including schizophrenia patients (*n* = 26) diagnosed on the basis of the Diagnostic and Statistical Manual of Mental Disorders (DSM)-IV criteria^14^. Samples from schizophrenia patients were obtained from the National Institute of Child Health and Human Development (NICHD) Brain and Tissue Bank for Developmental Disorder at the Maryland Psychiatric Research Center, The Harvard Brain Tissue Resource Center, and the University of Maryland^14^. This lipidomics study identified a total of 5024 lipids^14^. Comparison of lipidome alterations revealed that 10.4% of the detected lipids (522/5024) were altered significantly in schizophrenia samples versus samples from cognitively healthy controls^14^. Furthermore, the authors analyzed the genetic variants associated with schizophrenia using the well-accepted genome-wide associations studies (GWAS), and identified the relationship between schizophrenia-associated genetic variants and lipids with decreased concentrations in schizophrenia subjects versus cognitively healthy controls^14^. Interestingly, these genes were found to be involved in 19 molecular pathways, some of which [e.g. glycerophosphoinositol (PI) signaling pathway] have been shown to be closely related to schizophrenia. Alterations in fatty acid concentrations were detected in the PFC of patients with schizophrenia^19^. In addition, it has been recently reported that sphingolipids and *N*-acyl-phosphatidylserine (NAPS) were elevated in the frontal cortex of individuals with schizophrenia, based on a study in which brain samples from schizophrenia patients and controls were obtained from the National Institute of Health (NIH) Neurobiobanks^20^. In assessments of membrane fluidity, an important parameter affected by membrane lipidome changes that characterizes physiological properties, it was found that membrane fluidity was reduced in schizophrenia samples compared with samples from cognitively healthy controls. These important findings about the genes related to lipids that are decreased in schizophrenia suggest that lipidome alterations in the frontal cortex gray matter in the brain may play an important role in the development and progression of schizophrenia^6–14^.

### Lipidomics of the retina and the correlation with schizophrenia

The retina represents a very complex neurosensory tissue comprised of multiple neuronal cell types. The blood-retina barrier (BRB) has been shown to participate in the transportation of some lipid species from the circulation to the brain^21^ and reportedly functions in parallel with the blood–brain barrier (BBB) to transfer docosahexaenoic acid (DHA) in the circulation to the brain^21^. Two separate BRBs exist in the retina and have distinct blood supplies^22–25^. The outer BRB is composed of tight junctions between the retinal pigmented epithelial (RPE) cells, while the inner BRB consists of tight and adherent junctions between adjacent endothelial cells through which circulating nutrients, including lipids, are transferred into the inner cell layers of the retina leukocytes^22–25^. Previous studies of retinal lipids and lipid metabolism were conducted mainly to address their assembly on the membrane and turnover in the rod and cone photoreceptor cells (PRCs). More recent studies of retinal lipids and lipid metabolism have focused on investigating disease-specific lipid species in the retina, which are pertinent to the composition and metabolism of lipids, as well as lipid-associated signaling in the retina.

Acar et al.^26^ performed lipidomic analyses of the retinas, optic nerves, and red blood cells (RBCs) obtained from healthy human donors. Liquid chromatography (LC) in combination with an electrospray ionization (ESI) source-MS (LC-ESI-MS) revealed that lipid entities in the RBCs were significantly correlated with representative pools of DHA and very-long chain PUFAs (VLC-PUFA) in retinal or optic nerve tissues^26^. Rice et al.^27^ conducted an in-depth investigation on the mechanism underlying the uptake, retention, and elongation of DHA in the retina, and identified a novel role for adiponectin receptor 1 (AdipoR1) as a regulatory switch that controls the DHA lipidome in PRCs and RPE cells. Interestingly, AdipoR1 ablation leads to a reduction in retinal DHA, disturbance of DHA elongation, and functional impairment as a result of retinal degeneration^27^. The levels of the identified individual species of lipids in RBCs may reflect their levels in the retina and optic nerve tissues, and they may represent good circulating biomarkers of ocular lipids.

It has been noted that patients with schizophrenia have a higher incidence of retinal abnormalities compared to healthy individuals. However, retinal alteration and schizophrenia could have a two-way relationship. From the existing data, we are inclined to think that retinal alteration onset may occur prior to the episode of schizophrenia. We also cannot exclude the possibility that retinal abnormalities could be worsened in patients with schizophrenia.

**Fig. 1:**
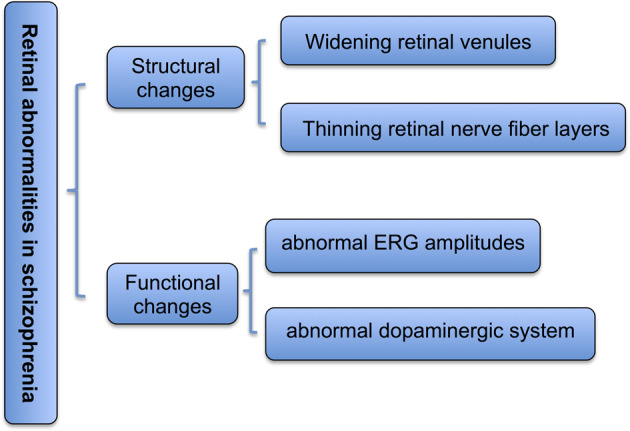
Structural and functional impairments of the retina in schizophrenia. The main retinal abnormalities in schizophrenia were schematically illustrated, including structural and functional impairments of the retina.

Electroretinography (ERG) tests, also referred to as electroretinagrams, have detected reduced a-wave or b-wave activity in individuals with schizophrenia^28,29^. The structural and functional impairments of the retina in schizophrenia are illustrated in Fig. 1. Hebert et al.^30^ reported that the cone a-wave amplitude, rod b-wave amplitude, and mixed cone-rod b-wave amplitude were significantly different between schizophrenia patients and control individuals. Notably, the findings obtained from this study with a large sample of schizophrenia patients were independent of the levels of medications or tobacco use^30^, suggesting a direct association between the impairment of retinal function at the levels of rod and cone photoreceptors and schizophrenia^30,31^. With the existing data, it remains unclear whether the retinal abnormalities could reflect parallel impairments in later visual processing and the brain function in schizophrenia. Several mechanism-of-action studies have proposed that a reduction in omega-3 fatty acids in the retina could be responsible for retinal abnormalities in the ERG test, and this hypothesis is supported by several lines of evidence, including lower levels of omega-3 fatty acids in schizophrenia, the effectiveness of omega-3 fatty acids in the treatment of schizophrenia, and the adverse effects of omega-3 fatty acid depletion on the function of the retina and indices in the ERG test^32–34^. Although further investigation on this issue is needed, it is increasingly apparent that parallel alterations in omega-3 fatty acids exist in retinal abnormalities and schizophrenia. Lavoie et al.^28,29^ also proposed that retinal abnormalities in the ERG test could reflect alterations in some transmitters (e.g. dopamine), in parallel with the changes in the brain. However, whether schizophrenia-specific lipids exist in the retina and whether there is an association between the altered lipids and schizophrenia have not been explored.

### Lipidomics of biofluids in schizophrenia

Previous studies have investigated lipids in biofluids [e.g. blood, cerebrospinal fluid (CSF), urea, and plasma, serum] in patients with schizophrenia in comparison to control subjects and have identified schizophrenia-related lipid abnormalities^12,32,35–37^. Many of these studies were performed to investigate the membrane lipids in RBCs, as they are considered to represent the tissue lipid status due to several features^26,38–40^. First, the lipids in RBCs are less sensitive to dietary fluctuations than those in the plasma or serum. Second, the lipids in RBCs represent a relatively longer-term individual status for the fatty acids. Third, the DHA levels in RBCs are closely correlated to the concentrations in neural tissues (e.g. optical nerve in the retina)^26,38–40^. A recent lipidomic study was performed to identify abnormal lipids on the membrane of RBCs in correlation with schizophrenia^12^. In human studies, Tessie et al. found that some membrane lipids on RBCs were altered in patients with schizophrenia compared with those in control individuals. Further animal studies have indicated that the altered lipid metabolism is correlated with the impairment of dopamine dysfunction^12,32^. Cai et al. also investigated the lipoproteins in plasma and urine samples from first-episode neuroleptic-naive schizophrenia (FENNS) patients. They found that the levels of various lipoproteins, including high-density lipoprotein (HDL), low-density lipoprotein (LDL), and very-low-density lipoprotein (VLDL), phosphatidylcholine (PC), phosphatidylethanolamine (PE), and unsaturated fatty acids (UFA) were decreased, while the levels of lysophosphatidylcholines (LPCs) in FENNS were increased in comparison with those in control individuals^41^. In agreement with the study by Cai et al. elevated levels of LPCs were also observed in RBCs and plasma samples from schizophrenia patients versus control individuals in another study^42^. Most recently, De Almeida and colleagues assessed alterations in the blood plasma lipidome in schizophrenia patients before and after treatment with atypical antipsychotics, and noted that some PCs were increased in response to antipsychotics^43^. It is worth noting that the differences in lipids between schizophrenia and control subjects in previous studies are inconsistent. For example, decreased LPC levels were reported in serum samples from twin pairs discordant for schizophrenia compared with unaffected healthy twin pairs as control subjects^10^. In contrast to the findings of reduced plasma lipoproteins in drug-naïve schizophrenia subjects versus control individuals, Tsang et al.^44^ reported elevated levels of lipoproteins in female schizophrenia patients who were monozygotic twins in comparison to the levels in the unaffected co-twins and healthy control subjects. Unlike the findings of reduced levels of PC and PE in the RBCs and plasma of patients with schizophrenia^8,41,42,45^, Lautin et al. reported no significant differences in the levels of the phospholipids PE, PC, and phosphatidylserine (PS) in RBCs between schizophrenia patients taking medications and healthy controls^46^. Similar results were reported in a study of first and recurrent-episode schizophrenia patients and control individuals^9^.

Compared with RBCs, the CSF is considered to more directly represent lipids in the brain since the main metabolites in the brain can diffuse into the CSF. However, there is a major limitation in lipidomics studies of the CSF because extraction of the CSF requires an invasive lumbar puncture that is dependent on considerable skill. As such, the study of lipid changes in RBCs, serum, and plasma samples is more convenient, less expensive, and safer.

Although different findings regarding aberrations in lipids between schizophrenia and control subjects exist in the literature and further studies are needed for confirmation, these studies suggest potential biomarkers for schizophrenia could include EPUFAs, lipid peroxidation metabolites, PC, and PE.

### Common aberrations of the lipidome in association with retinal abnormalities and the pathogenesis of schizophrenia

**Fig. 2:**
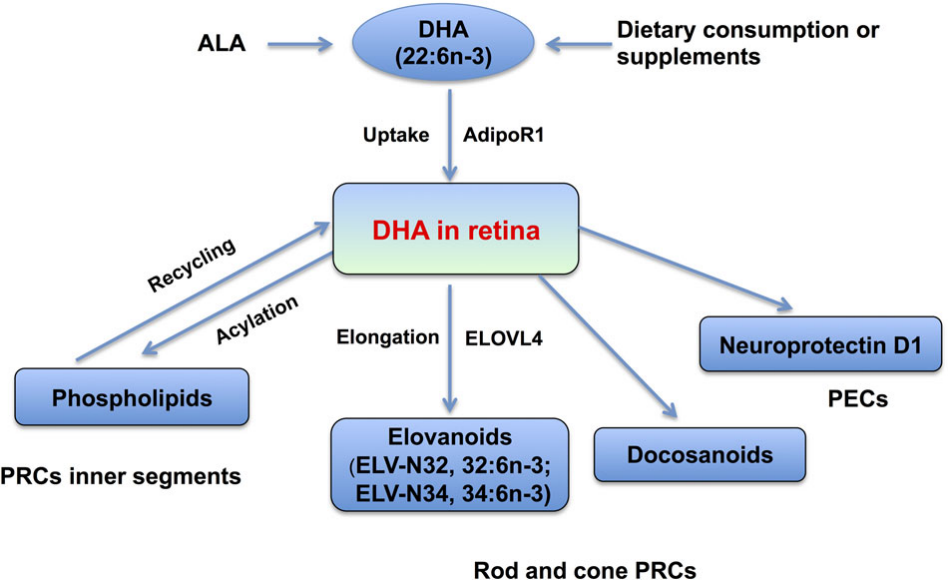
Schematic diagram of uptake and metabolism of main omega-3 fatty acids in the retina. The sources of DHA include the conversion of alpha-linolenic acid (ALA) into DHA in the liver, dietary consumption, and supplements. ALA as the most common dietary omega-3 fatty acid is converted to eicosapentaenoid acid (EPA, 20C, 5 double bonds), and then to docosahexaenoic acid (DHA, 22C, 6 double bonds) in the liver. The uptake and metabolism of DHA in the retina are schematically illustrated. The uptake of DHA occurs in the pigment epithelial cells (PECs) as well as in cone and rod retinal photoreceptor cells (PRCs), during which adiponectin receptor 1 (AdipoR1) captures DHA.

**Fig. 3:**
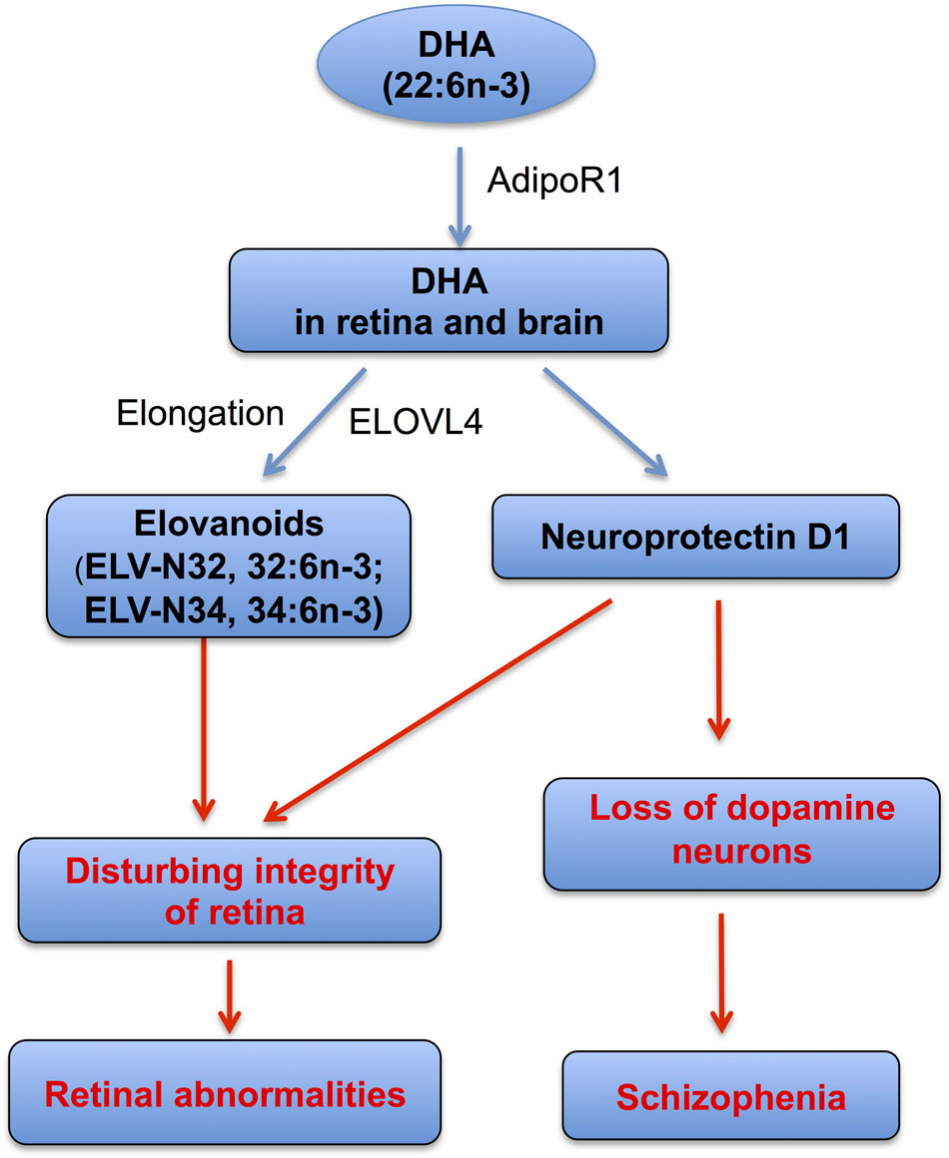
Common dysregulation in the uptake and metabolism of main omega-3 fatty acids and derivatives in retinal abnormalities and early events of schizophrenia. Dysregulation in the uptake and metabolism of omega-3 fatty acid docosahexaenoic acid (DHA) and its derivatives in the retina and the brain are associated with retinal abnormalities and early events in the pathogenesis of schizophrenia. Adiponectin receptor 1 (AdipoR1), primarily found in the eye and brain with little expression in other tissues, captures DHA in both the retina and brain. DHA is an important precursor of VLC-PUFAs (32:6n-3 and 34:6n-3), which subsequently turn into elovanoids (ELVs, ELV-N32, and ELV-N34) catalyzed by the elongase enzyme, specifically through the elongation of very-long-chain fatty acids-4 (ELOVL4). In addition, DHA is the precursor of neuroprotectin D1 (NPD1). Both ELVs and NPD1 sustain the integrity of the pigment epithelial cells (PECs) as well as photoreceptor cells (PRCs) in the retina. Dysregulation of DHA uptake and conversion into ELVs and NPD1 can disrupt the structural and functional integrity of the retina, leading to retinal abnormalities. In the brain, NPD1, converted from its precursor DHA, is involved in the protection of dopaminergic circuits. Dysregulation of DHA uptake and its conversion into NPD1 is associated with the loss of dopamine neurons, which is proposed to contribute to the pathogenesis of schizophrenia.

Previous studies of lipidomics in the brain and retina identified common aberrant alterations of lipids and lipid metabolisms, and the findings provide multiple lines of evidence in support of the concept that aberrations in some lipid species (e.g. phospholipids and PUFAs) lead to structural alterations and, in turn, impairments in the biological function of proteins bound to the membrane, disruption of cell signaling molecule accessibility, and dysfunction of neurotransmitter systems (e.g. dopamine). Based on the existing data, the dysregulation of lipid metabolism for specific molecules (e.g. DHA and phospholipids) in the retina is proposed in relation to the pathogenesis of schizophrenia. This hypothesis is supported by multiple lines of scientific evidence. The uptake of DHA is highly selective, with predominant uptake in the retinal cone and rod PRCs as well as in the brain. The outer segments of PRCs, along with the cellular membranes in the CNS, have been shown to contain the highest concentrations of DHA among all tissues. Interestingly, AdipoR1 is primarily expressed in the retina and brain, with little expression in other tissues^27,47^. AdipoR1 captures DHA in the retina and brain, and the uptake of DHA in the brain is similar to that in the retina. The uptake and metabolism of DHA in the retina is schematically illustrated in Fig. 2. It is proposed that the dysregulation in the uptake and metabolism of DHA and its derivations is associated with retinal abnormalities and early events in the pathogenesis of schizophrenia (Fig. 3)^27,47^. Notably, AdipoR1 ablation leads to a reduction in retinal DHA, disturbance of DHA elongation, and functional impairment as a result of retinal degeneration. These findings suggest that AdipoR plays an essential role in the structural and functional integrity of the retina^27^. DHA serves as an important precursor of VLC-PUFAs (32:6n-3 and 34:6n-3), which are then converted into elovanoids (ELVs, ELV-N32, and ELV-N34) catalyzed by the elongase enzyme with the elongation of very-long-chain fatty acids-4 (ELOVL4). DHA is also the precursor of neuroprotectin D1 (NPD1). ELVs and NPD1 have been shown to sustain the integrity of PECs and PRCs. Dysregulation of DHA uptake and conversion into ELVs and NPD1 can disrupt the structural and functional integrity of the retina and cause retinal abnormalities^27,47–49^. In the brain, NPD1 exerts a protective effect on dopaminergic circuits and disruptions to DHA uptake and its conversion into NPD1 may lead to the loss of dopamine neurons and may contribute to the pathogenesis of schizophrenia.

These proposed mechanisms could be highly helpful in improving our understanding of the pathogenesis of schizophrenia and for the design of novel therapeutics for schizophrenia. The newly identified DHA-signaling pathways could lead to addressing the important questions underlying the pathogenesis of schizophrenia and identifying novel molecular targets in the development of potentially therapeutic neuroprotective interventions in schizophrenia. These unveiled molecular targets may include AdipoR1 for the selective promotion of the DHA uptake, retention, and elongation by the retina and the brain, docosanoids (e.g. NPD1), and ELVs, with protective effects against retinal abnormalities and the loss of dopamine neurons (Fig. 3).

Common aberrations of the lipidome in association with retinal abnormalities and the pathogenesis of schizophrenia have important clinical implications. Supplementation with the main essential omega-3 fatty acids, including ALA, DHA, and EPA, may warrant consideration for the prevention and treatment of schizophrenia and retinal abnormalities, especially at the early stage of these disorders in clinical practice.

## Implications, perspectives, and challenges of the aberrant lipidome for decoding the mechanisms and developing new markers for schizophrenia

### Implications, perspectives, and challenges of the aberrant lipidome for decoding the mechanisms

**Fig. 4:**
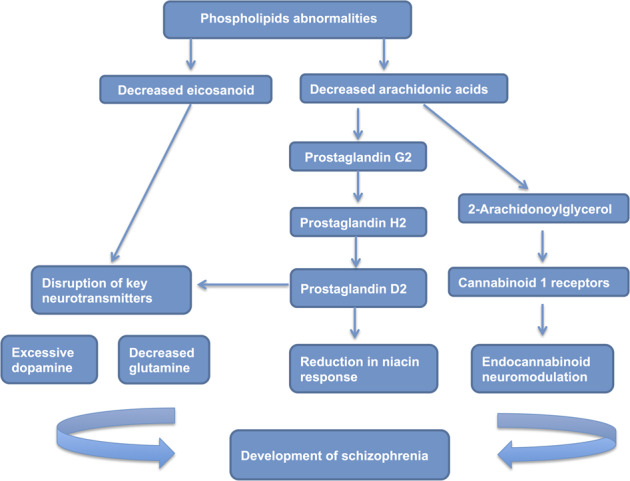
Involvement of lipids, mainly phospholipids and polyunsaturated fatty acids, in the pathogenesis of schizophrenia in the brain. Dysregulation in phospholipids and the metabolism may drive critical events involved in the development of schizophrenia.

As lipids possess numerous important biological functions, such as neurotransmission, signal transduction, and receptor binding^50^, recent findings regarding some lipid species and genes related to lipids that are significantly altered in schizophrenia suggest that lipidome alterations may play an important role in the development and progression of schizophrenia^6–14^. Furthermore, these schizophrenia-associated lipids and aberrant lipid metabolism may have important implications and perspectives for decoding the mechanisms of schizophrenia (Fig. 4). These lipids are as follows: (1) PUFAs. Abnormal alterations of PUFAs have been associated with schizophrenia^50,51^. As PUFAs are essential in neurodevelopment, disruption of the PUFA metabolism has been proposed to be involved in the pathogenesis of neurodevelopment disorders, such as schizophrenia and autism. For instance, Pawelczyk et al.^51^ found that first- episode schizophrenia patients prior to receiving antipsychotic therapy experienced an interruption in the PUFA metabolism, mainly with decreased levels of DHA and arachidonic acid (AA) in the frontal lobes of the brain, suggesting that PUFAs are associated with the onset of schizophrenia. The levels of AA have been consistently reported to be lower in patients with schizophrenia versus control individuals, and the alteration of AA is consistent in the literature^50,51^. Most recently, Messamore and Yao showed a link between altered AA and a sub-type of schizophrenia^52^. The reduced levels of PUFAs in schizophrenia therefore suggest the therapeutic potential of some PUFA species in the disease^53^; (2) Phospholipids. It is well accepted that normal phospholipid metabolism is essential for the normal architecture and function of the brain. Phospholipids play important roles not only in maintaining the normal structure but also in multiple biological functions of the neurons. Abnormal alterations in phospholipids have been reported to diminish the function of ion channels, neurotransmitters, and cell signaling^54,55^. In a study of schizophrenia, Horrobin and Bennett^56^ suggested that neurotransmitter-related abnormalities in schizophrenia are, at least in part, attributable to phospholipid abnormalities in the neurons. Reduced levels of phospholipids have been consistently reported in patients with schizophrenia, including drug-naïve and neuroleptic-naïve schizophrenia patients^8^. In a recent study, Messamore and Yao^52^ demonstrated a direct link between abnormal levels of phospholipids and abnormal neurochemicals, such as schizophrenia-associated abnormal dopamine and glutamate. These findings support the concept of aberrations in the phospholipid metabolism, mainly enhanced activity of phospholipase A_2_ (PLA_2_), and decreased integration of PUFAs into the phospholipids. These aberrations in phospholipid metabolism result in alterations in the structure of the membrane and consequently in the biological function of proteins bound to the membrane, disruption of cell signaling molecule accessibility, and dysfunction of neurotransmitter systems^57–59^. This assumption is supported by a number of previous studies indicating that (1) schizophrenia is associated with alterations in membrane-bound proteins for lipid transport and the composition of phospholipids (e.g. decrease in PE and PC, increase in PS); (2) an association exists between PLA2 and the dopamine system; (3) PL metabolism plays critical roles in the growth and remodeling of synapses as well as its dysfunction in relation with abnormal neurodevelopment in schizophrenia^57–59^.

### Implications, perspectives, and challenges of the aberrant lipidome for developing new markers for schizophrenia

Recent years have witnessed increasing interest and effort in the development of non-invasive, objective biomarkers for the diagnosis of diseases including schizophrenia. With regard to lipids, the above findings suggest their role potential biomarkers for schizophrenia including phospholipids (e.g. PC, PE), essential PUFAs (EPUFAs), and lipid peroxidation metabolites^60^. For example, He et al.^45^ established a set of plasma markers [glutamine, histidine, arginine, ornithine, phosphatidylcholine acyl-alkyl C38:6 (PC ae C38:6)], and lipids were included in the panel. These biomarkers in combination achieved an area under the curve (AUC) of 0.805. Thus, the abnormal schizophrenia-specific lipids in biofluids (Table 1) have important clinical implications and provide new perspectives regarding the aberrant lipid metabolism for the development of new markers for schizophrenia.

**Table 1:** Potential biomarkers identified in lipidomics studies of biofluids from schizophrenia patients.

Methods for lipidomics	Lipid species identified by lipidomics	Biofluids	References
UPLC-ESI- QTOF-MS	Triglycerides (lipid cluster, LC4 to LC9)	Serum	Orešič et al.^10^
UPLC-ESI- QTOF-MS	Lysophosphatidylcholines		Orešič et al.^10^
HPLC-ELSD and GC-FID	Triacylglycerols, free fatty acids, phosphatidylcholine, phosphatidylethanolamine	Plasma	Kaddurah-Daouk et al.^8^
TLC and GC-FID	Phosphatidylcholine (n3, n6), phosphatidylethanolamine (n3, n6)		McEvoy et al.^9^
ESI-MS/MS	Choline plasmalogen, ethanolamine plasmalogen, docosahexaenoic acid		Wood et al.^13^
UPLC-ESI- QTOF-MS	Free fatty acids, ceramide	Red blood cells	Schwarz et al.^6^
ESI-MS/MS	Choline plasmalogen, ethanolamine plasmalogen, docosahexaenoic acid	Platelets	Wood et al.^13^

### Challenges for potential clinical applications as new markers for schizophrenia

Despite the exciting findings in biological research, the following challenges have been identified for future research from the biological landscape towards potential clinical application of these aberrant lipid species as potential biomarkers for schizophrenia: (1) Schizophrenia-associated lipidome changes have overlaps with those in other neuropsychiatric disorders, such as autism, thereby posing challenges in the differential diagnosis of schizophrenia from other neuropsychiatric disorders; (2) Inconsistencies in the aberrations in lipid species between schizophrenia versus control subjects exist in the literature, leading to difficulties in identifying schizophrenia-specific lipids that can be used in the development of biomarkers for the diagnosis of schizophrenia; (3) There are technique-related challenges. For example, MS-based lipidomics methods require relatively large amounts of samples for the extraction of lipids for lipidomics studies; (4) Interpreting lipidomics data remains difficult, since the data generated in lipidomics is much larger and detailed. Among these, the large disparities across the technologies and methodologies used by different research groups may represent the most important challenge for the potential clinical application of lipids as new markers of schizophrenia. Thus, a standard is urgently needed. Fortunately, an effort has been initiated by Lipidomics Standards Initiative (LSI) to facilitate the standardization of lipidomics and to establish standards in all aspects of lipidomics spanning lipid analysis, pre-analytics, lipid extraction methods, MS, analysis of data, quality controls, and minimal reporting^61^.

## Conclusions

Lipidomics has become a new tool for exploring the pathomechanisms of schizophrenia and a repository of promising biomarkers has been identified for the early detection and diagnosis of schizophrenia based on some lipid species altered in biofluids. Further studies are required to validate these findings across research teams and cohorts. Despite the promise of the findings, it has been noted that little consensus between studies has been reached, mainly due to variability across laboratories worldwide and the relatively small size of the samples in existing studies. The establishment of a lipidomics workflow or platform that can yield consistent results across laboratories will be an important step towards potential clinical application of these lipid biomarkers. Furthermore, future studies will be needed to explore blood-based lipidomics changes and neuropathology, and correlate the lipidomic changes with genomic and proteomic alterations in schizophrenia. It has to be pointed out that inconsistencies in the aberrant alterations across laboratories exist in the literature; therefore further investigations will be needed to validate important findings from lipidomics studies.
